# The Impacts of Binge Drinking and Hangover on the Social Brain: An Integrative Narrative Review

**DOI:** 10.3390/biomedicines13112802

**Published:** 2025-11-17

**Authors:** Zsolt Bagosi, Gergely Karasz, Attila Ágoston Thury, Balázs Simon, Imre Földesi, Krisztina Csabafi

**Affiliations:** 1 Department of Pathophysiology, Albert Szent-Györgyi Medical School, University of Szeged, 6720 Szeged, Hungary; cl6kari@gmail.com (G.K.); csabafi.krisztina@med.u-szeged.hu (K.C.); 2 Department of Radiology, Albert Szent-Györgyi Medical School, University of Szeged, 6720 Szeged, Hungary; thuryati@gmail.com; 3 Department of Oncoplastic and Burn Surgery of Military Hospital Medical Centre, 1134 Budapest, Hungary; simonbazsi@gmail.com; 4 Institute of Laboratory Medicine, Albert Szent-Györgyi Medical School, University of Szeged, 6720 Szeged, Hungary; foldesi.imre@med.u-szeged.hu

**Keywords:** binge drinking, hangover, social brain, neurohormones, neurotransmitters

## Abstract

Binge drinking is defined as consuming a large amount of alcohol in a short period of time, whereas hangover is a cluster of unpleasant mental symptoms and physical signs that typically manifest the next day after binge drinking. Binge drinking is a prevalent pattern of alcohol consumption, especially in adolescents, with dualistic effects on social behavior. While some studies demonstrate that a single episode of binge drinking enhances sociability and preference for social novelty, other studies indicate that repeating cycles of binge drinking and hangover can lead to persistent negative affect and consequently social withdawal. This is an integrative narrative review synthesizing human studies and animal models of binge drinking (also known as alcohol intoxication) and hangover (also known as alcohol withdrawal). The major databases consulted were PubMed, Scopus, and Web of Science. The search terms used were “binge drinking” or “hangover”and “social behavior” or “social brain” in combination with “rats”, “mice” or “humans”. Finding the missing link between structural and functional changes in the social brain in the context of binge drinking and hangover is crucial for developing novel therapeutic strategies for alcohol intoxication and withdrawal. This review focuses on changes in hypothalamic neurohormones and extrahypothalamic neurotransmitters in these states, and concludes with the statement that targeting neuropeptides such as corticotropin-releasing hormone (CRH) and arginine vasopressin (AVP) and their receptors, which are involved in both binge drinking and social behavior, may prevent repeated cycles of binge drinking and hangover from spiraling into alcohol addiction and, ultimately, social isolation.

## 1. Introduction

### 1.1. Definition of Binge Drinking

Binge drinking is consuming a large enough amount of alcohol within a short period to raise the blood alcohol concentration (BAC) to 0.08% or higher [1]. This large amount of alcohol corresponds to five or more standard drinks for men, and four or more standard drinks for women, and the short period refers to two hours [1]. However, the alcohol content in a standard drink varies widely between countries, ranging from 8 to 20 g [2]. Based on epidemiological data, binge drinking is a prevalent pattern of alcohol consumption, especially in adolescents, with dualistic effects on social behavior [3,4]. Some studies demonstrate that binge drinking can enhance sociability and preference for social novelty, at least immediately after binge drinking [5]. Other studies demonstrate that repeating cycles of binge drinking and hangover can lead to persistent negative affect, and consequently social withdrawal [6]. Adolescents are particularly vulnerable to the effects of binge drinking because during adolescence the brain is still undergoing significant development, including brain regions involved in cognitive and affective processes [7,8]. Moreover, repeated episodes of binge drinking and hangover during adolescence can spiral into alcohol addiction during adulthood, which corresponds to a moderate-to-severe form of alcohol used disorder (AUD) which can affect the social life of men and women [9]. Nevertheless, men and women usually behave differrently under the influence of alcohol intoxication [10,11].

During binge drinking, men often lean towards competition, risk-taking (like drunk driving or consuming more alcohol), aggression (leading to verbal or physical fights), and a dangerous inclination towards sexual assault due to disinhibition and a false sense of entitlement [10,11]. In contrast, women tend to seek emotional intimacy and social validation, with disinhibition leading to open sharing of feelings and heightened displays of affection, but also an increased vulnerability to sexual assault and victimization by men [10,11].

### 1.2. Definition of Hangover

Hangover is a cluster of unpleasant mental symptoms and physical signs that typically manifest the next day after binge drinking when BAC aproaches or reaches 0% [12,13]. The symptoms of hangover usually start 6 to 8 h after binge drinking stops, and can last for up to 24 h [12,13]. Common symptoms of hangover include headache and dizziness, nausea and vomiting, thirst and hunger, anxiety and depression, irritability and hypersensitivity to light and sound, which can be explained by dehydration, gastrointestinal inflammation, hypoglycemia and insomnia, which are caused by alcohol and its toxic metabolites (e.g., acetaldehyde) [14,15,16,17]. In general, social interactions are reduced in this period due the social withdawal induced by the unpleasent mental symptoms and signs, but sexual desire can be increased or decreased because of fluctuations in sexual hormones, with different impacts on men and women [12,13].

During hangover, men predominantly seek solitude to manage physical discomfort and negative affect (“hangxiety”). Coping includes minimization with humor, exaggeration for empathy, ill-advised physical activity (“sweating it out”), or consumption of more alcohol (“hair of the dog”) [18]. In comparison, women typically experience greater physical discomfort and negative affect, leading to higher rates of sick leave or canceled engagements. Women are more sociable the next morning, using peer interaction to discuss their anxiety, especially concerning perceived social or sexual transgressions from the previous night [18].

### 1.3. Concept of Social Brain

**Figure 1:**
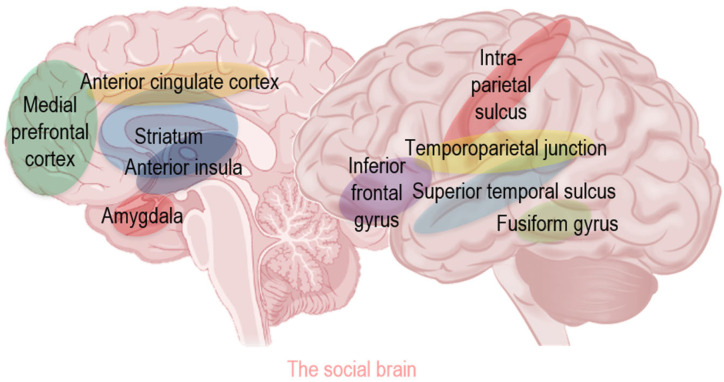
The social brain: a network of interconnected brain regions that regulate various aspects of social behavior [22].

From a simple gesture like a fleeting glance across the room at another person to the more complex social dynamics characterizing a long-term-relationship, social interactions are regulated by the “social brain” [19,20,21]. The social brain is not a single, anatomical structure, but includes a network of interconnected brain regions involved in various aspects of social behavior [20] (Figure 1).

For example, the medial prefrontal cortex, ventromedial cortex, and anterior cingulate cortex form a critical fronto-limbic circuit responsible for executive functions (planning, decision-making and impulse control) and emotional regulation (inhibiting fear/anxiety, processing guilt and detecting conflict/errors) [19,20,21]. The striatum serves as the core of the reward system, driving motivation for social connection, and is activated by social praise and cooperation. The anterior insula contributes to self-awareness and processes violations of social norms (e.g., disgust) [19,20,21]. Furthermore, the amygdala is central to emotion processing and interpreting social cues, while the temporoparietal junction, superior temporal sulcus, and fusiform gyrus are specialized in social perception, handling everything from distinguishing perspectives to processing biological motion and recognizing faces [19,20,21]. Finally, the inferior frontal gyrus is vital for emotional empathy and theory of mind, allowing individuals to attribute mental states to others and regulate their own impulses in complex social settings [19,20,21].

## 2. Methodology

### 2.1. Type of Review

This is an integrative narrative review synthesizing human studies and animal models of binge drinking (also known as alcohol intoxication) and hangover (also known as alcohol withdrawal).

### 2.2. Aim of the Study

Both human and animal studies on binge drinking demonstrate structural changes in brain regions, such as the prefrontal cortex, amygdala, hippocampus, and striatum, but the link between these changes and social behavior is still missing. Finding the missing link between the structural and functional changes to the “social brain” in the context of binge drinking and hangover is crucial for developing novel therapeutic strategies for alcohol intoxication and withdrawal.

### 2.3. Search Strategy

The major databases consulted were PubMed, Scopus, and Web of Science. The search terms used were “binge drinking” or “hangover”and “social behavior” or “social brain” in combination with “rats”, “mice” or “humans”.

### 2.4. Limitations of the Study

This review summarizes the results from studies in humans, rats and mice, but some of these studies only used adolescent subjects (no adults) or only males (no females), therefore analyses of the age- or sex-dependent changes in these cases were not possible. There are additional limitations due to small sample sizes, particularly in animal studies, and differences in the alcohol administration protocols, even between studies using the same paradigms (i.e., drinking in the dark procedure).

## 3. Human Studies

### 3.1. Clinical Tests

Binge drinking and hangover can clearly affect the cognitive and affective functions of the brain, impacting the social interactions between people. But the evidence for this is not merely anecdotal; the impacts on the body and the brain have been the subjects of rigorous clinical investigation [4,8].

A clinical investigation might start with registration of an electrocardiogram (ECG) and determination of blood pressure, since alcohol intoxication can lead to arrhythmias (“holiday heart syndrome”), myocardial damage (dilative cardiomyopathy) and increase in blood pressure (primary and secondary hypertension) [4]. Since low doses of alcohol can reduce rapid eye movement (REM) sleep, and high doses of alcohol may shorten sleep onset latency, determination of the sleep pattern by polysomnography in a laboratory setting or actigraphy in an ambulatory setting may prove useful [4].

Beyond these objective methods of investigation, there is a whole series of tests used to assess the cognitive and affective deficits caused by binge drinking [23,24]. For example, cognitive performance tests can evaluate higher-order cognitive processes, such as planning, decision-making, attention and working memory [23]. Among these, the Wisconsin card sorting test assesses cognitive flexibility and the ability to shift problem-solving strategies [25]. The stroop color–word test measures selective attention and inhibitory control [25]. Verbal fluency tests require participants to generate words within a specific category or starting with a particular letter, assessing executive function and verbal memory [25]. The Rey auditory verbal learning test and the California verbal learning test evaluate verbal learning and memory, whereas the Rey–Osterrieth complex figure test and the prospective memory video procedure investigate non-verbal memory [25]. Simple reaction time or choice reaction time tasks are used to investigate attention and speed of response to a stimulus, while sustained attention to response tasks are used to assess the ability to maintain attention on a stimulus over time [23].

Besides these objective tests, there are several subjective, self-reported methods used to quantify the symptoms and signs caused by hangover [26,27]. For example, on the acute hangover rating scale participants can rate the severity of their symptoms (e.g., headache and fatigue) on a numerical scale from 0 to 10 [26,27]. But the hangover symptom scale provides an even more comprehensive list of symptoms, which can be similarly rated [26,27]. Moreover, on visual analog scales participants can mark a point on a continuous line between two extremes to indicate the intensity of their symptoms (e.g., from “no headache” to “worst possible headache”) [26,27].

### 3.2. Laboratory Tests

The most important laboratory test is the determination of BAC, because it is included in the definition of both binge drinking and hangover, but its importance spans the entire spectrum of AUD, from the lab bench to the hospital bedside [4,8].

The methods used to determine BAC can be classified into direct and indirect measurements [28]. Direct methods are considered the gold standard in laboratory settings [28]. One of these methods is based on the chemical propensity of ethanol to be oxidized by a solution of potassium dichromate in an acidic environment [28]. The color change of the solution from orange to green is considered a positive test, and the intensity of the color is proportional to the ethanol concentration [28]. Another method is gas chromatography, in which a blood sample is prepared first, often using a technique called headspace analysis, and then the blood is injected into a gas chromatograph [28]. A gas cromatograph can separate the ethanol from other volatile components in the blood based on their different physical and chemical properties, and then a flame ionization detector quantifies the amount of ethanol present in the blood [28]. An enzymatic assay can be used to determine BAC directly from a blood sample [28]. In this method, alcohol dehydrogenase is used because it can oxidize ethanol in the presence of nicotinamide adenine dinucleotide (NAD^+^), producing acetaldehyde and a reduced form of nicotinamide adenine dinucleotide (NADH) [28]. The amount of NADH produced can be measured by spectrophotometry and is directly proportional to the amount of ethanol in the blood sample [28]. The indirect methods are less accurate, but also less invasive; they can determine BAC based only on the concentration of alcohol in breath, saliva, urine and sweat [28]. The best example for such methods is a breathalyzer that is used in roadside testing [28]. Actually, different types of breathalyzers exist, including those using fuel cell technology or infrared spectroscopy [28]. These devices convert breath alcohol concentration to an estimated BAC with a known (approximately 2100:1) ratio [28]. In animal research both methods can be applied, but gas chromatography and enzymatic assays are the methods primarily used [28]. Blood is collected from the trunk or from the tail of the animal, the latest alowing for repeated, serial measurements [28]. Breathalyzers can be also adapted for research animals, often by creating a chamber to collect breath samples, since this method is less disturbing for the animal [28].

Other possible markers of AUD could be liver enzymes, including alanine amino-transferase (ALAT), aspartate amino-transferase (ASAT), gamma glutamyl-transferase (GGT) and pro-inflammatory cytokines such as interleukin-1 (IL-1), interleukin-6 (IL-6) and tumor necrosis factor alpha (TNF-α) [29,30].

### 3.3. Paraclinical Tests

Binge drinking can cause structural and functional changes in the gray matter and white matter of the brain [8]. Changes in gray matter are detected usually by magnetic resonance imaging (MRI), which uses strong magnetic fields and radio waves to create images of the brain [8]. Changes in white matter are primarily detected by diffusion tensor imaging (DTI), a specialized MRI technique that measures the direction and magnitude of water molecule diffusion within the brain [8].

There are two types of MRI: structural and functional MRI. Structural MRI is used to capture detailed images of the brain [31]. The principle of this technique is based on the fact that a strong magnetic field aligns the protons in the body’s water molecules, and when radiofrequency pulses are applied, these protons are momentarily knocked out and then relaxed back to their original alignment [31]. The scanner measures the different relaxation times of protons in various tissues, providing a clear distinction between gray matter, white matter, and cerebrospinal fluid [31]. Therefore, a structural MRI scan can generate a static, but detailed image of the brain’s anatomy [31]. In contrast, functional MRI is used to measure brain activity in real-time [32]. The principle of this technique relies on the observation that when a brain region becomes more active, the body responds by increasing blood flow transporting more oxygen-rich hemoglobin to that region [32]. Since deoxygenated and oxygenated hemoglobin have different magnetic properties, the scanner can detect the subtle changes in magnetic signals and take a series of rapid images, creating a “brain map” of areas which are more and less active [32]. During the scan, the subject is usually asked to perform a specific task (e.g., a cognitive test), and thereby a task activation means an increase in activity, while a task inhibition refers to a decrease in activity in a certain brain region observed during that task [32].

DTI is a more advanced form of MRI that measures the diffusion of water molecules within the brain [33]. By measuring the direction of water diffusion, called fractional anisotropy, and the overall rate of water diffusion regardless of direction, called diffusivity, within the bundles of myelinated axons, this technique can infer the health and integrity of white matter tracts [33]. Lower fractional anisotropy values can indicate damage to axons or myelin sheaths, which disrupts the organized diffusion of water, whereas increased mean diffusivity can indicate an increase in the space between cells, such as from edema or neurodegeneration [33].

### 3.4. Results from Human Studies

**Figure 2:**
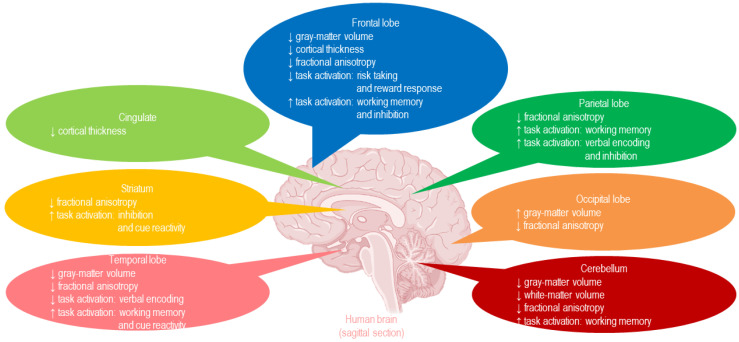
The impacts of binge drinking and hangover on the brain: human imaging studies primarily show structural changes in gray matter and functional changes in white matter in several cortical and subcortical areas. ↓ = decreased; ↑ = increased [8].

Human imaging studies have found reduced volume of gray matter in adolescents and young adults, explained by slowing down in the maturation of different areas (e.g., the prefrontal cortex vs. striatum) and by an accelerated decline in gray matter caused by premature exposure to alcohol [34] (Figure 2).

Among cortical structures, the prefrontal cortex, which is linked to executive functions like decision-making and impulse control, seems to be particularly affected, resulting in the poor judgment, increased impulsivity, and difficulties in problem-solving observed in adolescent binge drinkers [34]. Alcohol-induced damage to the parietal cortex, critical for visuospatial processing and attention, and to the temporal cortex, essential for auditory processing and language, can impair these cognitive functions and have impacts on social interactions [34]. Damage to the insula, which is involved in perception of the inner body and mental state, is associated with an increased risk of developing AUD and can impair one’s ability to interpret internal cues such as the negative effects of intoxication [34]. Reduced gray matter volume in the anterior cingulate cortex, which plays a particular role in the regulation of cognition and emotions, can contribute to the compulsive behaviors and emotional outbursts of anger or joy associated with binge drinking [34].

Binge drinking can also affect subcortical structures such as the striatum, the amygdala and the hippocampus [34]. While some studies have reported gray matter loss in the striatum, others have described a paradoxical increase in size that is associated with the transition from recreational drinking to habitual use of alcohol [34]. Decreased volume and increased reactivity in the function of the amygdala while watching pictures of alcoholic beverages or emotional faces may explain the emotional deficits observed in binge drinkers [34]. Moreover, smaller hippocampal volume may explain cognitive deficits, as it is directly linked to the memory blackouts, concentrating and learning difficulties often experienced by binge drinkers [34].

In addition, microstructural and functional changes in white matter have been observed, particularly in the corpus callosum, the main bridge connecting the two brain hemispheres, as well as in frontal, parietal, and temporal white matter tracts [34]. These changes can disrupt the brain’s ability to integrate information (e.g., between the prefrontal cortex and amygdala), leading to deficits in both cognitive and emotional empathy and repercussions on social life [34] (Table 1).

A consistent finding across functional MRI studies performed in binge drinkers is the disruption of the frontostriatal circuit that connects the prefrontal cortex with the NACC and CP, and this finding may explain the why binge drinking becomes in time an impulsive act [52]. Functional MRI also suggests decreased connectivity between the globus pallidus and the striatum in impulsive binge drinkers, which may lead to the brain’s inability to signal a stop to drinking [53]. Functional MRI studies suggest that there is compensatory over-activation in the inhibitory control network, specifically the inferior frontal gyrus and anterior insula, when successfully trying to suppress binge drinking [53]. Moreover, positron emmision tomography (PET) imaging consistently demonstrates a reduction in D2 receptor (D2) availability in the striatum of individuals with AUD, which is associated with greater impulsivity and correlates negatively with the severity of alcohol craving [54]. It seems that binge drinkers must expend increased cognitive effort to exert inhibitory control over impulsive behavior [54].

**Figure 3:**
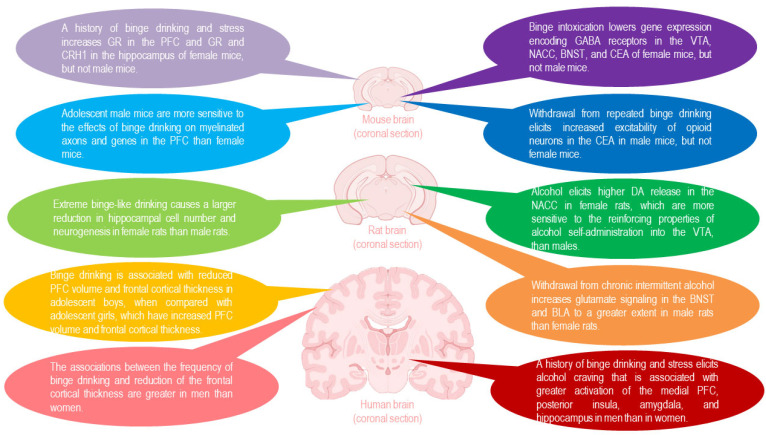
The impacts of binge drinking and hangover on the brain: age- and sex-dependent differences are seen across species, including in mice, rats and humans [10].

In summary, human studies have demonstrated that the brains of men are more vulnerable to the impact of binge drinking compared to women, and that adolescents are more sensitive to both the positive, rewarding properties and the negative, adverse effects of alcohol, and therefore, more susceptible to developing alcohol addiction, than adults [55,56,57,58,59,60,61,62,63,64,65,66]. Age- and sex-dependent differences in the structure and/or function of brain regions, such as the prefrontal cortex, hippocampus, VTA, NACC, BNST, CEA and BLA were also confirmed in mice and rats [34] (Figure 3).

## 4. Animal Models

### 4.1. Administration Protocols

The ethical and practical limitations of human studies may require the use of animal models. Animal models of binge drinking and hangover primarily involve mice and rats [67]. The greatest challenge of these models is to convince animals to voluntarily drink alcohol and to achieve physiologically relevant BAC at the same time [67]. Consequently, the animal models of binge drinking can be divided in two main categories: voluntary oral self-administration models, which better reflect the voluntary nature of human alcohol consumption [68], and forced administration models, which allow better control over BAC [69].

The “Drinking in the dark” procedure, originally described by Rhodes, belongs to the first category [70]. This method is based on the observation that some inbred strains of mice (e.g., C57BL/6 or DBA/2 mice) voluntarily drink alcohol, especially in the active (dark) phase of their circadian cycle [68]. The most common variation of this procedure uses singly housed mice; their circadian cycle is first inverted for 14 days, and then their water bottles are replaced with bottles containing 20% alcohol for 2 to 4 h during their dark phase [70]. However, in order to reach consistently high BACs, this procedure is sometimes modified: sometimes different access periods are applied, sometimes access to a singe bottle or two bottles (water and alcohol) are allowed so animals are allowed to choose between an alcohol solution and water, and sometimes drinking periods are alternated with periods of abstinence (e.g., access every other day) [71]. In most of these paradigms the concentration of alcohol ranges between 5, 10, 20 and 40%, but the highest BACs are reached by consuming 20% alcohol solution [71].

“Chronic intermittent ethanol exposure” belongs to the second category of procedures and its first description is attributed to Majchrowicz [72]. In this paradigm, outbred strains of rats (e.g., Sprague–Dawley or Wistar rats) are used, which normally do not drink alcoholic beverages [69]. Therefore, alcohol is usually delivered by intragastric gavage, which can be repeated three or four times within a day, similarly to the classical method of intraperitoneal (IP) injection of alcohol [73,74]. Both the concentration (10–30%) and total volume (10–20 mL/kg) of alcohol are critical to achieving the desired BAC, but once reached, the BAC can be maintained quite constant [75]. The periods of alcohol intoxication can last for 4 or more days, and can be followed by 1 or more days of alcohol withdrawal, resembling the states of binge drinking and hangover [73,74].

### 4.2. Behavioral Tests

The social interactions of rodents can be investigated by behavioral tests that are simple or more complex, specific for different aspects of social behavior, such as sociability and preference for social novelty, social recognition and memory, aggressive and affiliative behavior and maternal and juvenile play behavior [76,77].

The simpliest test to investigate social behavior is the reciprocal social interaction test described by File et al., which requires an open arena and allows direct interactions between two rodents [78]. In this test, the subject animal lies in its home cage or a novel cage-sized arena and then the stimulus animal is introduced [79]. The following behaviors are assessed: nose-to-nose or oral-to-oral, nose-to-genital or nose-to-anal sniffing, one animal following or chasing another, one animal pouncing or pinning another, crawling under or over each other (sexual behavior), and lunging or biting each other (aggressive behavior) [79]. In addition, the social play behaviors characterizing juvenile rodents and including pouncing or nape attacks, wrestling or tumbling, chasing or fleeing during play, can be also observed in a novel arena, especially after a certain period in which the animals were separated, as social isolation enhances the play drive [80].

The three-chamber social interaction test invented by Crawley et al. is more complex and more specific: it can assess the sociability and preference for social novelty of rodents without interfering with aggressive or sexual behavior [81]. In this case, the arena consists of three chambers that are separated by two removable Plexiglass walls [82]. In the first phase of the test (habituation), the subject animal is put in the middle chamber and allowed to freely explore the entire arena and get used to the novel environment [82]. In the second phase of the test (sociability test), a stranger rodent is placed in one of the side chambers within a wire mesh enclosure, while an empty, identical enclosure is placed in the opposite side chamber [82]. By removing the two mobile plexiglass walls, the subject animal is allowed to explore the adjacent chambers [82]. In the third phase of the test (preference for social novelty test), the empty enclosure is replaced with one that includes a second stranger, while the original stranger, considered now familiarized, remains in its enclosure [82]. Again, the subject animal is allowed to explore the adjacent chambers [82]. In both tests, the number of entries to the stranger are counted and the time spent in interaction with the stranger are measured [82].

A modified version of the three-chamber social interaction test is the two-chamber social interaction test developed by Moy et al. [83]. In this test, the arena is divided into two equally sized compartments by a Plexiglas wall with an aperture in the middle to allow movements of the rodents between the two compartments [84]. In this test, the subject animal is first habituated to the arena, and then a social stimulus animal is introduced in the other compartment [84]. The social investigation frequency and the social preference/avoidance coefficient are measured [84]. Social investigation is recorded when the subject animal is sniffing any part of the body of the social stimulus animal [84]. Social preference/avoidance is indexed when the experimental animal moves toward or away from the social stimulus animal [84].

The social recognition memory test described by Thor and Holloway involves familiar and stranger stimulus rodents, and investigates more specifically the cognitive aspect of social behavior [85]. In the first phase of this test (familiarization), the test animal is exposed to a “familiar” and usually juvenile stimulus rodent for a certain period, and in the second phase of the test (regognition), after a certain delay, the test animal is presented with both the familiar rodent and a novel “stranger” rodent [86]. The time spent investigating the novel stranger rodent versus the familiar rodent is measured as an index for social recognition memory [86].

The resident–intruder test is preferred when the aim is to investigate aggressive and submissive behaviors in rodents [87]. This test was described in detail by Koolhaass et al. [87]. First, the “resident” male rodent is singly housed in its home cage for a period to establish territoriality and sometimes also coupled with a female rodent [87]. Then an unfamiliar, “intruder” male rodent (often smaller than the resident male rodent) is introduced into the resident’s cage, so the resident animal will inevitably attack the intruder [87]. In this test, the latency to the first attack and the frequency and duration of offensive behaviors (e.g., lunging, biting and chasing the intruder by the resident) and defensive behaviors (e.g., upright posture, lying on back and avoiding the resident by the intruder) are registered [87].

### 4.3. Results from Mouse Studies

Mouse studies are based on the observation that some inbred strains of mice (e.g., C57BL/6 or DBA/2 mice) voluntarily drink alcohol, especially in the active (dark) phase of their circadian cycle [67]. In one of these studies, adolescent male C57BL/6 mice were exposed to a drinking in the dark procedure for 4 days and investigated in an elevated plus-maze test, a forced swim test and a three-chamber social interaction test. The study demonstrated that alcohol produced anxiolytic and antidepressant effects immediately after binge drinking, which were reduced by the selective corticotropin-releasing hormone (CRH) receptor type 2 (CRH2) antagonist astressin2B, but not the selective CRH receptor type 1 (CRH1) antagonist antalarmin [5]. Moreover, alcohol enhanced the sociability and preference for social novelty of mice immediately after binge drinking [5]. But the picture changes significantly after 24 h, since mice exposed to alcohol presented anxiety-like and depression-like signs, which were reversed by antalarmin but not astressin2B [5]. These signs of negative affect typically appear within 8–10 h and can last until 24 h, and correspond with the state of hangover [88,89]. Hangover is usually associated with social withdrawal, but mice exposed to alcohol did not show any significant change in social interaction 24 h after binge drinking [5]. This observation was supported by another study using C57BL/6 mice of the same age and sex, according to which mice expressed signs of negative affect, particularly anxiety-like behavior, but no change in sociability or preference for social novelty after 1 day and 21 days of alcohol withdrawal [6,90,91]. The drinking in the dark procedure often involves singly housed adolescent animals, and social isolation can inherently lead to anxiety and increase alcohol intake compared to group-housed animals [92,93]. Interestingly, while several studies indicated that group housing and exposure to physical exercise of adolescent mice protect against cognitive, emotional and social deficits in adulthood [94,95], one study demonstrated that social isolation of adolescent mice results in the development of a hypersocial phenotype in adulthood [96]. However, binge drinking can also cause escalations in aggression, the level of which may depend on the amount of alcohol [92,93]. When adult male CFW mice were pair-housed with females for at least 2 weeks to facilitate territorial behavior toward male conspecifics, repeated cycles of binge drinking caused increased frequency of attack bites during resident–intruder tests during acute withdrawal, but this aggressive behavior was observed only in high drinkers (with 1.33 g/kg/h alcohol intake), not in low drinkers (with 0.45 g/kg/h alcohol intake) [92,93]. Moreover, animals exposed to 5 weeks of binge drinking initiated fights more rapidly and with greater consistency after 1 day, and this aggressive behavior was still found following 1 week of abstinence [92,93]. The knockout of dopamine (DA) receptor type 4 (D4) reduced the sociability of adolescent male, but not female mice, and decreased aggression in adolescent female, but not male mice, after two weeks of abstinence. Therefore, it was suggested that D4 could mediate affiliative behavior in males and aggressive behavior in females [97]. Moreover, binge drinking can also cause reduction in ability to recognize familiar conspecifics [92,93]. Alterations in social recognition could be mediated by serotonin (SER) receptor type 2c (5HT2c), since 5HT2c deficient female but not male mice showed reduced social recognition after 7 days following repeated cycles of binge drinking [98]. In general, when both adolescent and adult, male and female DBA/2 mice were investigated 1 day after repeated cycles of binge drinking that corresponded with the state of hangover, a significant reduction in social interactions was observed in adult, but not adolescent mice, and no differences between male and female mice were reported [99].

Overall, studies in mice suggested that the negative impact of binge drinking and hangover on social behavior might not always be present after a single episode of alcohol intoxication, but may manifest during acute alcohol withdrawal following several cycles of binge drinking, with possible differences between males and females [6,90,91] (Table 2). The negative impacts were linked to damage to the ventral tegmental area (VTA), the NACC, the bed nucleus of stria terminalis (BNST), the central nucleus of the amygdala (CEA) and the basolateral nucleus of the amygdala (BLA) [73]. Imbalances in several neuropeptide systems, such as CRH and urocortin 1 (UCN1), arginin vasopressin (AVP) and oxytocin (OXY), and classical neurotransmitters such as DA and SER, gamma amino-butyric acid (GABA) and glutamate (GLU) were also implicated [73].

### 4.4. Results from Rat Studies

In contrast with mice, rats do not drink alcohol voluntarily, unless it is sweetened with sucrose or it has a low alcoholic content (e.g., beer) [72]. Thereby, in order to meet the BAC criteria for binge drinking, higher doses of alcohol must be administered via IP injection or intragastric gavage [73,74]. In earlier studies, male Wistar rats were exposed to chronic intermittent alcohol exposure by intragastric gavage, followed by a battery of behavioral tests, including novel object recognition and social discrimination tests [100,101]. Binge-like drinking induced deficits in novel object recognition and social discrimination in adult male rats 3 days after the last administration of alcohol, indicating a significant cognitive and social deficit [100,101]. Later, high-alcohol-preferring rat strains, such as Sardinian rats, have been developed from Wistar rats, which readily consume large amounts of alcohol, often preferring it over water [102,103]. Consequently, studies using adult, male and female Sardinian alcohol-preferring rats suggested that social interactions are reduced in adult rats 12 h after binge drinking, but this reduction dissipates 24 h after binge drinking, and no differences between males and females were noted [104,105,106]. Still, differences arising from the age or sex of the animals must be taken into consideration, because it can influence many aspects of social behavior. For example, male rats showed altered sensitivity to acute alcohol challenges, exhibiting facilitation of social investigation and play fighting, which is reminiscent of adolescent behavior [107,108,109,110,111]. In contrast, female rats showed little alteration in sensitivity to acute alcohol challenges regarding measures of social investigation and play fighting, independently of the dose of alcohol or time of exposure [107,108,109,110,111]. Moreover, the impacts of binge drinking on social behavior may depend on the dose and the timing of alcohol intake [101,112]. While low concentrations can induce social facilitation, particularly in adolescent rats, high concentrations lead to social inhibition in both adolescent and adult rats [83]. Besides a general reduction in social interaction, the sociability and preference for social novelty of rats were also tested [107,108,109,110,111]. In order to do so, adolescent and adult, male and female Sprague–Dawley rats were exposed to chronic intermittent alcohol by intragastric gavage, followed by plus-maze test and a two-chamber social interaction test, a modified version of the three-chamber social interaction test [107,108,109,110,111]. In males, but not females, reductions in social investigation and social preference was observed after 2 days, which persisted after 25 days following the last alcohol administration [107,108,109,110,111]. Further studies with male and female cFos-LacZ transgenic rats with the same Sprague–Dawley background consistently reported that intermittent alcohol exposure during adolescence results in long-lasting social impairments during adulthood, particularly in males, with no apparent effect on females [107,108,109,110,111,113,114,115,116].

Overall, studies in rats have confirmed what the mouse studies suggested, that social deficits may manifest during acute alcohol withdrawal (after 12 h) and may persist during protracted alcohol withdrawal (after 21 days), with possible differences between males and females [73] (Table 3). Beyond the brain regions and mediators already shown to be involved, the role of the extended amygdala (CEA-BNST-BLA) and that of ghrelin and opioids were emphasized [73].

## 5. Discussion

**Figure 4:**
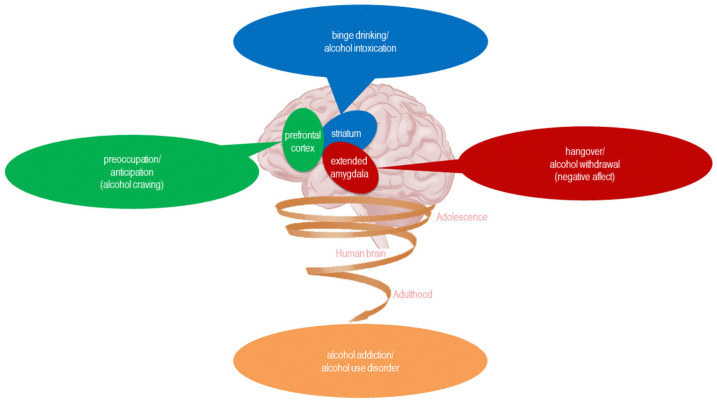
The impacts of binge drinking and hangover on the brain: repeated cycles of binge drinking and hangover during adolescence can spiral into alcohol addiction during adulthood [34].

Repeating cycles of binge drinking and hangover during adolescence may spiral into alcohol addiction later in adulthood. Alcohol addiction classically progresses through three stages: binge drinking (also known as alcohol intoxication), hangover (also known as alcohol withdrawal) and craving (also known as preoccupation/anticipation). Each stage is characterized by specific changes in hypothalamic neurohormones, such as CRH, UCN1, AVP and OXY, and extrahypothalamic neurotransmitters, such as DA, SER, GABA and GLU [119] (Figure 4).

### 5.1. Hypothalamic Neurohormones

#### 5.1.1. Corticotropin-Releasing Hormone (CRH) and Urocortin 1 (UCN1)

**Figure 5:**
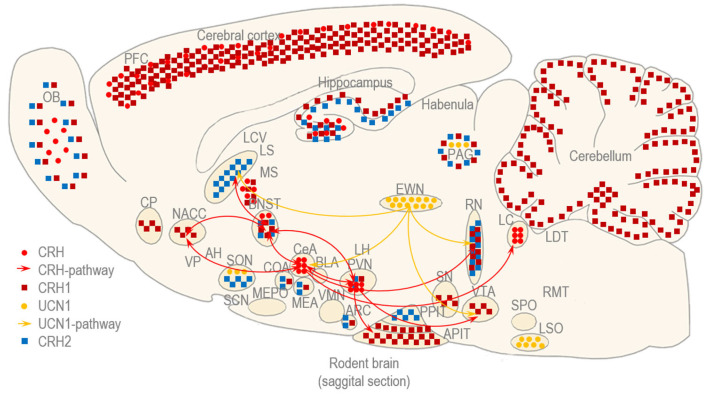
The CRH and UCN1 system: stress-related neurohormones such as CRH and UCN1 are involved in binge drinking and hangover, and some of their pathways and receptors can mediate social behavior.

CRH is the major hypothalamic neurohormone that orchestrates the neuroendocrine, autonomic and behavioral responses to stress [120,121]. It is a 41 amino acid neuropeptide that acts via two G-coupled receptors: CRH1 and CRH2, with putatively antagonistic actions in the central nervous system (CNS) [122]. CRH1 is distributed predominantly in the brain, in the cerebral cortex, anterior pituitary and cerebellum, and less represented in the periphery [123] and promotes stress, anxiety and depression [120,121,124]. In contrast, CRH2 is more abundant in the periphery, and limited centrally to the subcortical regions, including the striatum, amygdala, and hippocampus [123] and mediates anxiolytic and antidepressant effects [120,121,124] (Figure 5).

CRH can act a hypothalamic neurohormone, regulating the activity of the hypothalamic–pituitary–adrenal (HPA) axis, but also as an extrahypothalamic neurotransmitter, mediating the processes linked to the extended amygdala [125,126]. As a neurohormone, CRH is synthesized and released from the PVN of the hypothalamus, and, after reaching the median eminence, it stimulates the secretion of adrenocorticotrophic hormone (ACTH) in the anterior pituitary, which in turn stimulates the secretion of corticosteroids in the adrenal cortex [125]. The increase in corticosteroid level in the blood (mainly cortisol in humans and corticosterone in rodents) not only reflects the activation of the HPA axis, but exerts negative feedback effects on the hypothalamic CRH and pituitary ACTH, turning down the stress response [125]. Moreover, CRH appears to modulate its own secretion by amygdala and peri-paraventricular GABA release [127]. As a neurotransmitter, CRH is produced abundantly in the CEA, connecting it with the BNST and the shell of the NACC. These structures are known together as the extended amygdala [126]. CRH can also stimulate noradrenaline (NA) release in the locus coeruleus (LC) and inhibit the SER release in the raphe nuclei (RN), along with UCN1 [128].

CRH seems to be involved in all stages of alcohol addiction [122]. The stage of binge drinking/alcohol intoxication is associated with the activation of the HPA axis that is initiated by hypothalamic CRH [129]. The stage of withdrawal/negative affect is associated with the activation of the extended amygdala circuit that is mediated by extrahypothalamic CRH and NA [129]. The stage of preoccupation/anticipation or craving is associated with the activation of the hippocampus, orbitofrontal cortex, prefrontal cortex, insula and BLA and believed to be mediated by both hypothalamic and extrahypothalamic CRH, which makes one vulnerable to relapse, especially in periods of stress [130,131]. CRH is involved in different aspects of social behavior as well, including aggressive and affiliative behavior, parental care, maternal defense, sexual behavior and pair bonding. For example, administration of sub-anxiogenic doses of CRH into the NACC facilitates partner preference formation in monogamous species, such as male prairie voles [132], but administration of anxiogenic doses of CRH in the lateral cerebral ventricle of non-monogamous species, including mice and rats, induces freezing and inhibition of all types of social interactions [133]. Furthermore, a markedly different distribution of CRH1 and CRH2 was described in monogamous vs. non-monogamous voles, involving brain regions such as the NACC and the lateral septum (LS) [133].

Since CRH was first isolated in the ovine hypothalamus, new CRH-like peptides called urocortins have been described. They have similar amino acid structures, but different anatomical distribution, physiological functions and pharmacological profiles compared with CRH [134,135,136]. While CRH binds preferentially to CRH1 and UCN1 binds with equal affinity to both CRH receptors, UCN2 and UCN3 bind with much higher affinity to CRH2; therefore, they are considered selective CRF2 agonists. But among the urocortins, it is UCN1 that presents the highest structural and functional homology with CRH [137].

UCN1 is expressed predominantly in the centrally projecting Edinger–Westphal nucleus (EWN), but also in the supraoptic nucleus (SON) of the hypothalamus [137] (Figure 5). Administration of UCN1 into the EWN leads to escalation of alcohol consumption in animal models of binge drinking, suggesting that it is involved in the first stage of alcohol intoxication [138]. Indeed, the EWN is part of a complex neural circuit that is highly sensitive to alcohol and other drugs of abuse, and it projects to areas like the RN, VTA, CEA and LS, which could be involved in the rewarding effects of alcohol by the release of extrahypothalamic neurotransmitters such as DA, SER, GABA and GLU [137].

Besides alcohol intoxication, UCN1 seems to participate in social interactions, but the results are contradictory in this sense: a single and repeated injection of UCN1 in the BLA and BNST in male rats decreased active social interaction time, while infusion of UCN1 in the lateral cerebral ventricle of male mice produced an increase in sociability (male–male interaction) and a decrease in the preference for social novelty (male–female interaction) [139]. This discrepancy can be explained by the fact that UCN1 expressed abundantly in the SON. may influence other hypothalamic neurohormones, such as AVP and OXY, resulting in complex social dynamics [140,141].

#### 5.1.2. Arginin Vasopressin (AVP) and Oxytocin (OXY)

**Figure 6:**
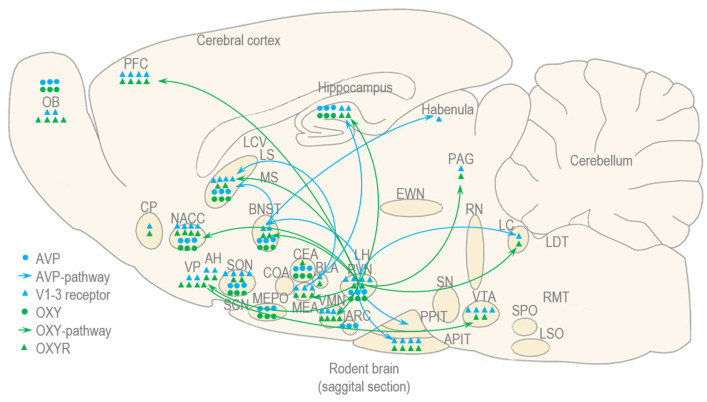
The AVP and OXY system: social neurohormones such as AVP and OXY are involved in binge drinking and hangover, and some of their pathways and receptors can mediate social behavior.

AVP, also known as antidiuretic hormone (ADH), is a 9-amino acid neuropeptide that is synthesized in the SON and PVN of the hypothalamus and can be involved in both binge drinking and social behavior, but its main physiological roles are regulation of water balance, blood pressure and stress response [142]. It exerts its effects via three different G-coupled receptors: vasopressin receptor type 1a (V1a), type 1b (V1b or V3) and type 2 (V2). The most well-known effects of AVP are the following: it increases water reabsorption in the kidney via V2 conserving water and maintaining osmolarity of the blood (due to osmotic activation of AVP); it constricts arterioles via V1a, increasing total peripheral resistance and blood pressure (due to non-osmotic activation of AVP); and it stimulates the release of ACTH in the posterior pituitary via V1b, activating the HPA axis, in synergy with CRH [142]. Besides its neuroendocrine function, AVP also plays a role in autonomic control and social behavior (e.g., pair bonding and territorial aggression), which are mediated by V1a and V1b receptors, expressed abundantly in the vasopressinergic projections of PVN and BNST [142] (Figure 6).

As the BAC rises, alcohol suppresses the release of AVP from the pituitary gland leading to increased diuresis [142]. The massive fluid and electrolyte loss (sodium, potassium, etc.) may contribute to the nausea, fatigue, headache and muscle weakness which are hallmark features of the hangover state [142]. As the BAC drops to zero, a “rebound” effect can occur where plasma AVP levels may be higher than normal as a physiological attempt to correct severe dehydration [142]. The fluctuations of plasma AVP levels during binge drinking and hangover can be related to changes in expression and concentration of hypothalamic AVP, which are matched by the plasma levels of ACTH and corticosteroids during alcohol intoxication and withdrawal [74].

As regards social behavior, AVP often works in conjunction with the related neuropeptide OXY [143]. Historically, AVP was viewed as mainly regulating male social behavior, while OXY was seen as regulating female behavior. However, current research shows that both peptides are involved in both sexes, with the effects of AVP often being more pronounced in males (e.g., social investigation and territorial aggression) and those of OXY being more evident in females (e.g., initiation of maternal behavior, mother-infant bonding) [143].

OXY and AVP share some anatomical and biochemical features, but OXY differs from AVP in its main physiological functions and pharmacological actions [143]. The major physiological functions of OXY are milk ejection and uterine contraction, which are mediated by a single G-coupled receptor, OXY receptor (OXYR), expressed in the periphery, in the myoepithelial cells of the mammary gland and uterus [143]. The pharmacological actions of OXY are pro-social and anxiolytic, demonstrated by intracerebroventricular and intranasal administration of OXY [143]. These actions are mediated by central OXY receptors expressed abundantly in brain regions associated with reward, such as the VTA and NACC and emotional processing, such as the CEA and the medial nucleus of the amygdala (MEA) [143] (Figure 6).

During binge drinking, OXY levels increase acutely in the blood, contributing to the subjective “feel-good” effects of alcohol, including the reduced social inhibition and enhanced social bonding that people experience while intoxicated [144,145]. But chronic heavy drinking and the state of hangover are characterized by lower OXY levels and/or decreased OXYR sensitivity that are often associated with negative affect, including increased stress reactivity, anxiety and social withdrawal, symptoms that may drive sober people to drink again [144,145]. Therefore, fluctuations of OXY levels in the blood may contribute to the cycle of alcohol addiction that has three stages, each stage being characterized by specific changes in extrahypothalamic neurotransmitters, such as striatal DA and brainstem SER, amygdala GABA and hippocampal GLU [74].

### 5.2. Extrahypothalamic Neurotransmitters

#### 5.2.1. Dopamine (DA) and Serotonin (SER)

**Figure 7:**
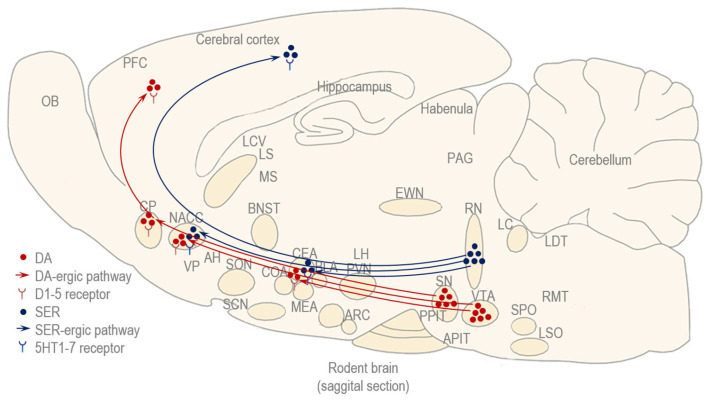
The DA and SER system: classical neurotransmitters such as DA and SER are involved in binge drinking and hangover, and some of their pathways and receptors can mediate social behavior.

DA is a monoamine, or more precisely, a phenethylamine derived from adrenaline and NA, and acts through five main types of receptors which belong to the D1-like family (D1 and D5 receptors) and are excitatory and coupled to G-stimulatory proteins (Gs) or D2-like family (D2, D3, and D4) receptors that are inhibitory and coupled to G-inhibitory proteins (Gi) [146]. There are two major DA-rgic pathways in the brain which mediate reward senzation and motor control: the mesolimbic pathway, which begins in the DA-rgic neurons of the VTA and ends in the ventral striatum and is represented mainly by the NACC; and the nigrostriatal pathway, which emerges in the substantia nigra (SN) and projects to the dorsal striatum, constituted by the CP [146]. The mesolimbic pathway also sends projections to the amygdala and the prefrontal cortex, therefore it is often referred to as the mesolimbicocortical pathway [146] (Figure 7).

During binge drinking, acute exposure to alcohol stimulates both the mesolimbic and nigrostriatal pathways, inducing striatal DA release, and produces the sensation of reward [147,148]. The rewarding effects of alcohol include an increased drive and motivation for social interaction manifested as increased sociability and preference for social novelty during binge drinking [149]. However, chronic alcohol exposure results in a reward deficit and activation of stress systems as an adaptation to repeated alcohol exposure [147,148]. The reward deficit can be caused by an increase in the reward threshold caused by the down-regulation of pre-synaptic DA receptors, and a decrease in extracellular DA release caused by the depletion of striatal DA stores [150]. The activation of the stress system includes activation of the HPA axis and that of the extended amygdala circuit, mediated by hypothalamic and extrahypothalamic CRH, respectively. Thereby, repeated cycles of binge drinking are characterized by a significant drop in DA levels, contributing to decreased drive and motivation for social interactions that typically manifest during the period of hangover [149].

SER or 5-hydroxy tryptamine (5HT) is an indolamine derived from the amino acid tryptophan, and acts through a diverse family of receptors, which can be divided into seven main types (5HT1 to 5HT7), and several subtypes within these classes, including the 5HT1 family (e.g., 5HT1A, 5HT1B and 5HT1D) that is Gi-coupled, and the 5HT2 family (5HT2A, 5HT2B and 5HT2C) that is Gq-coupled. SER is found both in the CNS and the periphery [151]. In the CNS, the major source of SER is the raphe nuclei (RN), including the dorsal and ventral group of nuclei, from where it is spread to many cortical and subcortical areas, mediating the feel-good sensation, learning and memory consolidation, sleep and pain [151]. Nevertheless, the majority of SER is found in the gut, from where it modulates gastrointestinal motility, secretions and appetite [151]. SER is also known to mediate proaffiliative and aggressive behavior [151] (Figure 7).

Binge-like consumption of alcohol was shown to stimulate the synthesis of SER in the RN, which can lead to a temporary boost in mood and appetite, and may even explain some social aspects of binge drinking, such as affiliation and aggression [149]. However, chronic consumption of alcohol leads to a depletion of SER, resulting in anxiety and depression, demasked during alcohol withdrawal [149]. Moreover, the SER depletion observed during periods of hangover can worsen pre-existing mood disorders and sleep disturbances and create a vicious circle where individuals may be tempted to drink again to ameliorate their negative affect, including the social withdawal experienced during hangover [149].

DA and SER do not operate in isolation, but in a dynamic, reciprocal fashion, often modulating each other’s activity [149]. While DA is linked to the “wanting” or appetitive aspect of social reward, SER can be seen as influencing the “liking” or avoiding of the social cues that might lead to unwanted social outcomes [149].

#### 5.2.2. Glutamate (GLU) and Gamma Amino-Butyric Acid (GABA)

**Figure 8:**
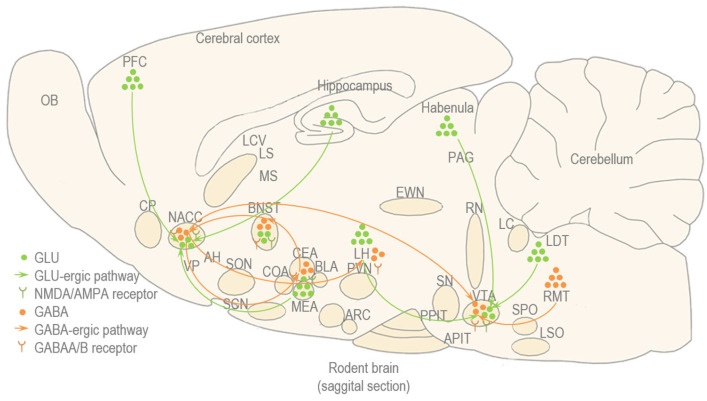
The GLU and GABA system: classical neurotransmitters such as GLU and GABA are involved in binge drinking and hangover, and some of their pathways and receptors can mediate social behavior.

GLU is the most abundant excitatory neurotransmitter in the brain, an amino acid that activate GLU acts through ionotropic receptors such as alpha-amino-3-hydroxy-5-methyl-4-isooxazole-propionic acid receptor (AMPA), receptors that are primarily permeable to sodium and potassium, and metabotropic receptors such as N-methyl-d-aspartate (NMDA) receptors, which are voltage-dependent and permeable to calcium [152]. The metabotropic GLU receptors include eight subtypes divided into three groups: group I (type 1 and 5), located postsynaptically and being excitatory, group II (type 2 and 3) and group III (type 4, 6, 7 and 8), located presynaptically and reducing GLU release and as such being inhibitory [152]. The major role of GLU is to stimulate neuronal activity, but it is also involved in synaptic plasticity, a process that is fundamental for cognitive functions such as arousal, learning and memory formation, but it also contributes to rapid communication between neurons throughout the brain and spinal cord, transmitting sensory information and motor functions [152]. In social contexts, the role of GLU is that of an accelerator, facilitating the rapid processing and integration of social cues [152]. The hippocampal GLU is presumed to play a role in arousal, learning and memory [149] (Figure 8).

Acute alcohol consumption decreases GLU-rgic neurotransmission in the hippocampus by down-regulation of NMDA and AMPA receptors, whereas chronic alcohol consumption increases GLU-rgic neurotransmission by up-regulation of the NMDA receptors and stimulation of GLU release, which might be further enhanced by repeated cycles of binge drinking and hangover [153,154,155,156]. As alcohol leaves the system, the brain, which has downregulated its GLU receptors in response to alcohol’s inhibitory effects, experiences a rebound in GLU activity, resulting in irritability and aggression, which can have detrimental effects on short-term interactions and long-term relationships [149]. These can explain the phenomenon of blackouts and the difficulties in interpreting social cues which can occur during alcohol intoxication, but also the irritability and aggresive behavior that are characteristic of alcohol withdrawal, each having their own detrimental effects on social interactions [149].

GABA is an amino acid synthesized from GLU, and represents the main inhibitory neurotransmitter in the brain [157]. GABA has two main types of receptors: GABA-A receptors that are ligand-gated chloride channels, so when GABA binds to them the channels open, allowing chloride ions to flow into the neurons and leading to hyperpolarization of the cell (fast synaptic inhibition); and GABA-B receptors that are G-coupled receptors, so when GABA binds to them, they activate inhibitory G-proteins or open potassium channels, leading to hyperpolarization of the cell (slow synaptic inhibition) or close calcium channels reducing the release of other neurotransmitters [157]. Overall, GABA exerts calming effects that are crucial for managing fear and anxiety and inducing and maintaining sleep [157]. GABA also helps in regulation of skeletal and smooth muscle tone and plays a role in coordinated movements and organized processing of sensory information by inhibiting unwanted neuronal firing [157]. In social contexts, GABA acts as a break, regulating emotional responses, particularly anxiety, and controlling impulsive or inappropriate social behaviors [157]. Amygdala GABA is presumed to play a role in the anxiolytic and sedative effects of alcohol [158,159,160,161] (Figure 8).

Acute alcohol consumption facilitates GABA-ergic neurotransmission in CeA via both pre- and post-synaptic mechanisms, whereas chronic alcohol consumption increases baseline GABA-ergic neurotransmission, but not stimulated GABA release [162]. During binge drinking, increased GABA activity can temporarily alleviate stress and anxiety, at least in the short term [149]. However, in the long-term, the CNS becomes accustomed to this increased GABA activity and leads to a down-regulation of GABA-A receptors and reduction of inhibitory signalling, such as the case of repeated cycles of binge drinking [149]. Moreover, GLU-rgic pathways involved in emotion are also regulated by GABA-ergic interneurons, hence the dysfunction of these can lead to emotional outbursts of exagerated joy or anger during alcohol intoxication [149]. The dysbalance between inhibitory and excitatory signaling in motor circuits of the brain can lead to a general hyperexcitability that manifests as termors and seizures during periods of alcohol withdrawal [149].

The balance between GLU-rgic excitation and GABA-ergic inhibition is critical for social functioning [149]. While GLU signalling promotes long-term potentiation, a process that strengthens synaptic connections essential for learning and social memory, GABA-ergic inhibition is crucial for shaping these plasticity events, ensuring precise and controlled learning in social contexts [149].

### 5.3. Translational Insights

The core limbic and frontal regions (e.g., striatum, amygdala and prefrontal cortex) of the social brain are consistently affected by binge drinking in both humans and rodents [7,8,34]. But human data directly confirms volume loss and specific cognitive, emotional and social deficits [8,34]. Both animal and human studies demonstrate that age- and sex-specific differences exist, but some studies have only investigated adolescent subjects (no adults) and males (no females), decreasing the translational value of these studies [10]. The activation of the stress system is stage-specific, but the distribution of stress-related neuropeptides (CRH and UCN1) is conserved in mice, rats and humans [133]. The distribution of social neurohormones (AVP and OXY) is also conserved across species, but their role in social behavior is highly specific to certain rodent models (e.g., voles) [133]. Finally, animal and human studies indicate that stage-specific changes in the major neurotransmitter systems (DA, SER, GLU and GABA) are highly conserved across species, providing better translational potential [7] (Table 4).

## 6. Conclusions

Binge drinking significantly disrupts the brain’s delicate neurochemical balance involving several key neurotransmitter (DA, SER, GLU, GABA) systems [163,164]. The positive, rewarding effects of alcohol are mediated primarily by the DA-rgic system, particularly the release of DA in the mesolimbic pathway, which drives the motivation for social interaction. Alcohol modulates the synthesis of SER in the RN and its release in projection areas such as the NACC, mediating affiliative and aggressive behavior. Concurrently, alcohol stimulates the release of inhibitory GABA and upregulates GABA-A receptors, especially in the amygdala, leading to an immediate calming effect and reduction of fear and anxiety. This inhibitory action is balanced by alcohol’s suppression of the excitatory GLU-rgic system, inhibiting receptors like NMDA in the hippocampus, contributing to blackouts during alcohol intoxication. Hangover is characterized by an attempt at neurochemical rebalancing [163,164]. The brain exhibits a period of excitability due to the body clearing alcohol, resulting in a compensatory down-regulation of GABA-A and an up-regulation of NMDA receptors. This imbalance between excitatory and inhibitory neurotransmitter systems induces a state of hyperexcitability that contributes to tremors and seizures primarily involving the prefrontal cortex and the limbic system. The DA-rgic and SER-ergic system is also affected, and the dysregulation of these neurotransmitters is associated with the mood disturbances such as anxiety, depression and social withdrawal experienced during hangover.

## 7. Future Directions

Binge drinking is a prevalent pattern of alcohol consumption, particularly during adolescence, with dualistic effects on social behavior [3,4]. While some studies demonstrate that binge drinking can enhance sociability and preference for social novelty, at least immediately after binge drinking [5], other studies demonstrate that repeating cycles of binge drinking and hangover can lead to persistent negative affect, and consequently social withdrawal [6]. Targeting neuropeptides such as CRH and AVP, and their receptors involved in both binge drinking and social behavior, may prevent repeated cycles of binge drinking and hangover from spiraling into alcohol addiction and, ultimately, social isolation. Therefore, central administration of CRH1 antagonists and/or CRH2 agonists may lead to reinstatement of the balance between the neurotransmitter (DA, SER, GLU and GABA) systems disrupted by binge drinking. This may lower alcohol intake during binge drinking and ameliorate the anxiety and depression experienced during hangover [165]. Furthermore, stimulating the central receptors of AVP (V1a and V1b) expressed in the brain and inhibiting the peripheral one found in the kidney (V2) may ameliorate the symptoms and signs of hangover, including social withdrawal [165]. Another remedy for hangover can be stimulation of OXYR by intranasal administration of OXY itself, due to its anxiolytic and pro-social effects [165]. Preclinical studies have already demonstrated the therapeutic efficacy of some of these peptides in rodents [165]. Despite the initial failure in translation of such therapy from animals to humans, future clinical trials should determine the effective dose and appropriate route of administration for these peptides in humans [166].

## Figures and Tables

**Table 1:** Studies in humans.

Brain Region	Structural Change	Functional Change	References
**Prefrontal cortex and** **inferior longitudinal fasciculus**	Thicker cortical thickness (gray matter), lower fractional anisotropy (white matter)	Poorer sustained attention, poorer working memory performance	Pfefferbaum et al., 2018 [35] and Bava et al., 2009, 2010, 2013 [36,37,38]
**Dorsolateral prefrontal cortex**	Greater gray matter volume	Increased working memory errors	Doallo et al., 2014 [39]
**Dorsolateral prefrontal cortex, inferior parietal lobule** **, d** **orsal cingulate and precuneu**	Smaller gray matter volume (dorsolateral prefrontal cortex and inferior parietal lobule) and greater gray matter volume (dorsal cingulate and precuneus)	Greater impulsivity	Xiao et al., 2013 [40]
**Left frontal pole and left pars orbitalis**	Thicker cortical thickness (in females) and thinner cortical thickness (in males)	Worse inhibition and attention and worse visuospatial construction (in females)	Squeglia et al., 2011, 2012, 2014 [41,42,43,44]
**Right middle anterior cingulate cortex**	Lower cortical thickness	Higher alcohol consumption	Mashhoon et al., 2014 [45]
**Ventromedial prefrontal cortex, right inferior frontal gyrus, left middle frontal gyrus, and right putamen**	Smaller grey matter volume (in ventromedial prefrontal cortex, right inferior frontal gyrus, left middle frontal gyrus and larger grey matter volume (right putamen)	Higher alcohol consumption	Whelan et al., 2014 [46]
**Ventral striatum**	Larger grey matter volume	Neuromaturational delay	Howell et al., 2013 [47]
**Fornix**	Reduced fractional anisotropy (white matter)	Predicted greater risky behavior 1.5 years later	Jacobus et al., 2013 [48,49,50,51]

**Table 2:** Studies in mice (↑ = increased; ↓ = decreased).

Animals	Methods	Results
Adolescent, male C57BL/6 mice	Drinking in the dark procedure, followed by elevated plus-maze, forced swim and three-chamber social interaction tests	↑ Sociability and preference for social novelty immediately after, but no change 1 day after a single cycle of binge drinking [5]
Adolescent, male C57BL/6 mice	Drinking in the dark procedure, followed by open-field, elevated plus-maze, forced swim, novel object recognition and three-chamber social interaction tests	No change in sociability and preference for social novelty 3 and 40 days after a single cycle of binge drinking [91]
Adolescent and adult, male and female DBA/2 mice	Drinking in the dark procedure, followed by novel object recognition test, open-field test and social interaction test	↓ Social interactions 1 day after, but no change 21 days after repeated cycles of binge drinking in adults, no change at all in adolescents, and no difference between males and females [99]
Adolescent, male and female C57BL/6 mice and D4 deficient mice with C57BL/6 background	Drinking in the dark procedure, followed by novel object recognition test, open-field test and social interaction test	↑ Social interactions in D4 deficient mice, but no change in females, and ↓ aggression in D4 deficient mice, but no change in males, 14 days after repeated cycles of binge drinking and [97].
Adolescent, male and female C57BL/6 mice and 5HT2c deficient mice with C57BL/6 background	Drinking in the dark procedure, followed by open-field, acoustic startle and social recognition test	↓ Social recognition in 5HT2c deficient females, but not males, 7 days after repeated cycles of binge drinking [98].

**Table 3:** Studies in rats (↓ = decreased).

Animals	Methods	Results
Adolescent male Wistar rats	Chronic intermittent alcohol exposure by intragastric gavage, followed by open-field test, plus-maze and conditioned place preference test or novel object recognition, social discrimination and conditioned place preference test	↓ Novel object recognition and ↓ social discrimination in adult males 3 days after the last episode of binge-like drinking [100,101]
Adult, male and female Sardinian alcohol-preferring rats with Wistar background	Drinking in the dark procedure, followed by social interaction test	↓ Social interactions in adults 12 h after, but no change 24 h after repeated cycles of binge drinking, and no difference between males and females [104,105,106]
Adolescent, male and female Sprague–Dawley rats	Acute and chronic intermittent alcohol exposure by intragastric gavage, followed by social interaction test	↓ Social interactions in males 1 day after repeated episodes of binge-like drinking, which persisted 21 days after, ↓ social interactions in females 1 day after a single episode or repeated episodes of binge-like drinking, which dissipated 21 days after the last epidose [117]
Adolescent and adult, male and female Sprague–Dawley rats	Chronic intermittent alcohol exposure by intragastric gavage, followed by plus-maze and a modified, two-chamber social interaction test	↓ Social investigation and social preference 2 days after the last episode of binge-like drinking, but only in males, not females, which persisted 25 days after [107,108,109,110,111]
Adolescent and adult, male and female cFos-LacZ transgenic rats with Sprague–Dawley background	Chronic intermittent alcohol exposure by intragastric gavage, followed by a modified, two-chamber social interaction test	↓ Sociability and social preference in adults, but only in males, not females, 25 days after the last episode of binge-like drinking [115,116,118]

**Table 4:** Parallels and Differences Between Rodents and Humans.

Feature	Rodents	Humans	Parallels and Differences
Structural changes	Animal models are primarily used to understand the neurobiological background of binge drinking and hangover. Studies often focus on the VTA, NACC, BNST, CEA, and BLA.	Human imaging studies show reduced volume of gray matter in the prefrontal cortex, amygdala, hippocampus, insula, anterior cingulate cortex and microstructural changes of the white matter in corpus callosum.	Parallel: core limbic and frontal regions are conserved targets for structural damage. Difference: human data show directly the volume loss that lie behind the cognitive and emotional deficits.
Functional changes	Animal models also help the understanding of the neurochemical background of binge drinking and hangover. Studies often focus on CRH, UCN1, AVP, OXY, DA, SER, GABA, GLU, ghrelin and opioids.	Human studies show that men are more vulnerable to the effects of binge drinking and hangover, than women, and adolescents are more susceptible to develop alcohol addiction, than adults.	Parallel: age- and sex-specific vulnerabilities exist across species. Difference: animal data show the neurobiological and neurochemical background of the cognitive and emotional deficits.
Stress-related neurohomones (CRH and UCN1)	HPA axis activation is involved in all addiction stages, reflected by elevated levels of ACTH and corticosterone in the blood. Activation of the extended amygdala circuit (CEA-BNST-NACC) is specific for alcohol withdrawal. Administration of CRH/UCN1 modulates partner preference in monogamous species (i.e., prairie voles).	HPA axis activation is involved in all addiction stages, reflected by elevated levels of ACTH and cortisol in the blood. The hyperactivity of the CRH system is indicated by the vulnerability to relapse, especially in periods of stress.	Parallel: The activation of the stress system is stage specific, but the distribution of CRH/UCN1 is conserved across species. Difference: The corticosteroids are primarily represented by corticosterone in rodents, and cortisol in humans.
Social neurohormones (AVP and OXY)	AVP mediates territorial aggression, whereas OXY facilitates pair bonding. Administration of AVP/OXY modulates partner preference in monogamous species (i.e., prairie voles).	Administration of AVP has anxiogenic effects, while OXY has anxiolytic and prosocial effects. Depletion of AVP induces water and electrolyte imbalance, while that of OXY contributes to social withdrawal during hangover.	Parallel: The distribution of AVP/OXY is conserved across species. Difference: The involvement of AVP and OXY in social behavior is highly specific to certain rodent models (e.g., voles).
Classical neurotransmitters (DA, SER, GABA, GLU)	Alterations correlate with animal behavior: DA depletion → reward deficit; SER depletion → depression-like behavior; decreased GLU neurotransmission and increased GABA neurotransmission → anxiety-like behavior.	Alterations correlate with human symptoms: DA depletion → craving; SER depletion → depression; GLU receptor upregulation → tremors and seizure; GABA receptor downregulation → fear and anxiety.	Parallel: The stage-specific changes of the major neurotransmitter systems (DA, SER, GLU, GABA) are highly conserved across species. Difference: Therapies targeting neurotransmitters are not always effective in both rodents and humans

## Data Availability

No new data were created or analyzed in this study. Data sharing is not applicable to this article.

## Abbreviations

The following abbreviations are used in this manuscript:

5HT1	5-hdroxytryptamine receptor type 1
5HT2	5-hdroxytryptamine receptor type 2
5HT3	5-hdroxytryptamine receptor type 3
5HT4	5-hdroxytryptamine receptor type 4
5HT5	5-hdroxytryptamine receptor type 5
5HT6	5-hdroxytryptamine receptor type 6
5HT7	5-hdroxytryptamine receptor type 7
ADH	antidiuretic hormone
ALAT	alanine amino-transferase
AMPA	alpha-amino-3-hydroxy-5-methyl-4-isooxazole-propionic acid receptor
ASAT	aspartate amino-transferase
AUD	alcohol use disorder
AVP	arginine vasopressin
BAC	blood alcohol concentration
BLA	basolateral nucleus of amygdala
BNST	bed nucleus of stria terminalis
CEA	central nucleus of amygdala
CNS	central nervous system
CP	caudate-putamen
CRH	corticotropin-releasing hormone
CRH1	corticotropin-releasing hormone receptor type 1
CRH2	corticotropin-releasing hormone receptor type 2
D1	dopamine receptor type 1
D2	dopamine receptor type 2
D3	dopamine receptor type 3
D4	dopamine receptor type 4
D5	dopamine receptor type 5
DA	dopamine
DTI	diffusion tensor imaging
ECG	electrocardiogram
EWN	Edinger-Westphal nucleus
GABA	gamma amino-butyric acid
GGT	gamma glutamyl-transferase
GLU	glutamate
ICV	intracerebroventricular
IP	intraperitoneal
IL-1	interleukin-1
IL-6	interleukin-6
LS	lateral septum
MEA	medial nucleus of amygdala
MRI	magnetic resonance imaging
NA	noradrenaline
NAD^+^	nicotinamide adenine dinucleotide
NADH	reduced form of nicotinamide adenine dinucleotide
NMDA	N-methyl-D-aspartate receptor
NACC	nucleus accumbens
OXY	oxytocin
OXYR	oxytocin receptor
Peri-PVN	peri-paraventricular nuclei
PET	positron emission tomography
PFC	prefrontal cortex
PVN	paraventricular nucleus
REM	rapid eye movement
SER	serotonin
SN	substantia nigra
SON	supraoptic nucleus
TNF-α	tumor necrosis factor-alpha
UCN1	urocortin 1
UCN2	urocortin 2
UCN3	urocortin 3
V1a	vasopressin receptor type 1a
V1b	vasopressin receptor type 1b
V2	vasopressin receptor type 2
VTA	ventral tegmental area
